# Lp-PLA_2_ activity and mass and CRP are associated with incident symptomatic peripheral arterial disease

**DOI:** 10.1038/s41598-019-42154-5

**Published:** 2019-04-04

**Authors:** Shahab Fatemi, Anders Gottsäter, Moncef Zarrouk, Gunnar Engström, Olle Melander, Margaretha Persson, Stefan Acosta

**Affiliations:** 1 Department of Clinical Sciences, Malmö, Lund University, Malmö, Sweden; 2 Department of Internal Medicine and Emergency Medicine, Skåne University Hospital, Malmö, Sweden; 3 Vascular Centre, Department of Cardiothoracic and Vascular Surgery, Skåne University Hospital, Malmö, Sweden

## Abstract

Long follow up is needed in prospective cohort study evaluation of plasma biomarkers for incident peripheral arterial disease (PAD) Middle-aged PAD-free individuals from the cardiovascular cohort of the Malmö Diet and Cancer study (n = 5550; 1991–94) were followed prospectively for a median time of 23.4 years. The plasma biomarkers lipoprotein-associated phospholipase A2 (Lp-PLA_2_) activity and mass, proneurotensin, and CRP, were studied in relation to incidence of PAD until December 31^st^, 2016. The diagnosis of PAD could be validated and confirmed in 98%. Cox regression was used to calculate hazard ratios (HR) per 1 standard deviation increment of each respective log transformed plasma biomarker. Cumulative incidence of PAD was 4.4% (men 5.9%, women 3.3%). Adjusting for age, gender, smoking, body mass index, hypertension, diabetes mellitus, Lp-PLA_2_ activity (HR 1.33; 95% CI 1.17–1.52), Lp-PLA_2_ mass (HR 1.20; 95% CI 1.05–1.37) and CRP (HR 1.55; 95% CI 1.36–1.76) remained independently associated with incident PAD. The plasma biomarkers Lp-PLA_2_ activity and mass, and CRP were markers of PAD risk, implying that they might be useful biomarkers for subclinical atherosclerosis and atherosclerotic disease.

## Introduction

Atherosclerosis is a systemic disease, often affecting the lower extremity arteries first^1^. Peripheral arterial disease (PAD), defined as occlusive atherosclerosis of the lower extremity arteries^2^ or the arteries distal to the aortic bifurcation, is highly associated with concomitant coronary, carotid and cerebral artery disease, an increased cardiovascular and overall mortality, despite secondary preventive efforts such as smoking cessation, antiplatelet and statin therapy^3^. Presence of PAD is associated with an extensive burden of atherosclerosis throughout the body and high, 29%, rate of silent myocardial infarctions^1^. Almost a fifth of elderly individuals have some stage of PAD and women are more afflicted than men^4^. The total number of individuals with PAD is peaking, with a 23% increase in the last decade due to increase in total population, global ageing, increased incidence of diabetes mellitus worldwide and smoking^5^.

Plasma biomarkers measured in healthy individuals may be useful to detect individuals at increased risk for developing PAD, and may also serve to identify factors enhancing or halting development of the disease. While C-reactive protein (CRP) is a well-established plasma biomarker for atherosclerosis^6^ and a predictor of worse outcome in patients with PAD^7^, several plasma biomarkers have been introduced in vascular research. The enzyme lipoprotein-associated phospholipase A2 (Lp-PLA_2_) is considered to be a biomarker for atherogenesis^8^. Lp-PLA_2_ levels have been associated with incident cardiovascular disease including coronary artery disease and ischaemic stroke^9^. The plasma biomarker proneurotensin^10^, a satiety regulator and marker of fat and digestion metabolism, has been found to be a associated with risk of cardiometabolic disease. Data on associations, however, between these biomarkers and incident PAD is scarce.

The main aim of this longitudinal cohort study was to evaluate Lp-PLA_2_ and proneurotensin in relation to an established risk marker such as CRP and known confounders for incident PAD risk at long term follow up.

## Material and Methods

### Study sample

**Figure 1:**
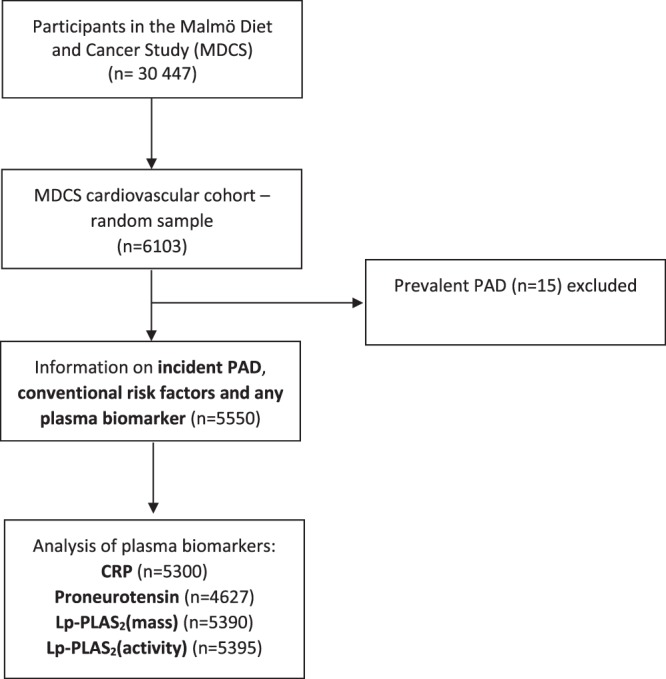
Descriptive flow diagram of study participants and plasma biomarker data. PAD; peripheral arterial disease, CRP; C-reactive protein, Lp-PLA2 (activity and mass); lipoprotein-associated phospholipase A2.

The population-based Malmö Diet and Cancer study (MDCS)^11–13^ included 30 447 middle-aged individuals from Malmö, Sweden. Participants attended baseline examinations between 1991–1996. Current smoking was defined as self-reported regular smoking or smoking cessation within the last year. Diabetes mellitus was defined as self-reported physician’s diagnosis, use of anti-diabetic medication, or fasting blood glucose >6.0 mmol/L. Hypertension was defined as use of antihypertensive medication or blood pressure ≥140/90 mmHg. From this cohort, a random sample, examined between November 1991 and February 1994 was included in the MDCS cardiovascular cohort^14^, of whom 5550 individuals underwent blood sampling under standardized fasting conditions^15^ (Fig. 1). Informed consent was obtained from all participants and long-term follow-up of study participants for incident cardiovascular disease. The Regional ethical review board in Lund, Sweden (Dnr LU 51/90; 2013/566) approved the study. All research was performed in accordance with relevant guidelines/regulations.

### Endpoint ascertainment

Individuals from the MDCS with a first registered diagnosis of PAD were identified from Swedish national registers^16^ (the Inpatient^17^ and Outpatient Register and the Cause of Death Register) by linkage of the ten-digit personal identification number unique to each Swedish resident. Follow-up extended until December 31^st^, 2016. In both the Inpatient and Cause of Death registers, diagnoses are coded using a Swedish revision of the International Classification of Disease (ICD), version 8 (443,90; 443,99; 440,20; 445,00; 445,98; 445,99), 9 (443×; 440 C), 10 (I73.9 [all sub codes except I73.9 A]; I70.2 [all sub codes]). Codes for embolism to the lower extremity (444,20, 444; I74.3, respectively) were excluded. Surgical procedures are coded using a Swedish classification system (Op6: 8884; 8885; 8815; 8816; 8817; 8818; 8886; 8887; 8825; 8826; 8827; 8828; 8861; 8862; 8865; 8866. KKÅ97: PDH 10; PDH 20; PDH 21; PDH 22; PDH 24; PDH 30; PDH 35; PDP 10; PDP 30; PDQ 10; PDQ 30; PDU 82; PDU 83; PDU 84; PDU 99; PEF (all sub codes); PEH (all sub codes); PEN (all sub codes), PEQ (all sub codes), PEU 82, PEU 83, PEU 84, PEU 99, PFH (all sub codes), PFN (all sub codes), PFP (all sub codes), PFQ (all sub codes), PFU 82; PFU 83; PFU 84; PFU 99; PGH 20; PGH 21; PGH 22; PGH 23; PGH 30; PGH 31; PGH 40.

### Validation of PAD diagnosis

One hundred patients with diagnosis of PAD were randomly selected for the validation procedure using patient record data. Among 100 patients, 69 had chronic limb-threatening limb ischemia, 13 had acute limb ischemia, 15 had claudication, and one had asymptomatic PAD. Among the 13 patients with acute limb ischemia, twelve had acute thrombotic occlusion and one had an embolic occlusion. Two patients had venous insufficiency and were misdiagnosed. PAD could therefore be confirmed in 98% of cases and symptomatic PAD in 97% of cases.

### Laboratory measurements

Plasma biomarkers were measured from fasting plasma samples that had been frozen at −80 °C immediately after collection^15^. Proneurotensin was measured using a chemiluminometric sandwich immunoassay to detect a proneurotensin fragment^10^. CRP was measured by a high-sensitivity Tina-quant^®^ latex assay (Roche Diagnostics, Basel, Switzerland). The average coefficient of variation (CV) was 4.59%^18^. Lp-PLA_2_ was expressed as enzymatic activity and mass (quantity)^19^. Lp-PLA_2_ activity was measured in duplicate using [3 H]-platelet activating factor as a substrate^19^. The range of detection was 8–150 nmol/min/ml. Samples were retested if the replicate CV was >20%. The average CV was 5.78%^18^. Lp-PLA_2_ mass measurements were performed using the second generation PLAQ^TM^ test (diaDexus Inc., South San Francisco, CA, USA) commercially available enzyme-linked immunosorbent assay (ELISA) kit^19^. All samples were analyzed in duplicate, and if a duplicate showed a CV of more than 20%, the sample was reanalyzed. The average CV was 4.62% on random of 50 first participants in the MDCS^18^. Plasma-EDTA samples are stable for Lp-PLA_2_ activity and mass measurements within 7 days of collection for refrigerated samples and for more than 10 years from collection when stored at −70 °C^19^.

### Statistical analysis

Quantitative normal and skewed distributed variables are presented as mean with standard deviation and median with interquartile range (IQR), respectively. Dichotomous variables are presented as count and proportion. Individuals with a diagnosis of PAD at baseline were excluded from the current study and prospective analyses included only incident PAD. Plasma biomarkers and confounders for incident PAD were assessed using Cox regression models, and hazard ratios (HRs) were expressed per one standard deviation (SD) increment of each respective log transformed plasma biomarker (skewed distributed) in the Cox regression models. Cumulative incidence of PAD was analyzed using Kaplan-Meier method. Log-rank test was used in the comparison of quartiles for Lp-PLA_2_ activity. Analyses were performed using SPSS for Windows, version 23.0 (SPSS Inc, Chicago, IL). A p-value less than 0.05 was considered significant.

## Results

### Baseline conventional risk factor assessment

Fifteen individuals with known PAD at baseline were excluded. The cumulative incidence of PAD was 4.4% (244/5550), 5.9% (137/2307) for men versus 3.3% (107/3243) for women (p < 0.001), during a median follow up period of 23.4 years (IQR 19.4–24.3). Baseline risk factor characteristics for individuals with or without PAD in the cohort are shown in Table 1. When including the conventional risk markers listed in Table 1 into a Cox regression analysis, age at baseline (p < 0.001), male gender (p < 0.001), current smoking (p < 0.001), diabetes mellitus (p < 0.001) and hypertension (p = 0.001) were independently associated with incident PAD, whereas BMI (p = 0.74) were not.

**Table 1:** Baseline characteristics in participants with and without later development of PAD.

Characteristic	No PAD (n = 5306)	PAD (n = 244)
Baseline age, years, mean (SD)	57.5 (5.9)	59.3 (5.6)
Male sex, %	40.9 (n = 2170)	56.1 (n = 137)
Body mass index, kg/m^2^, mean (SD)	25.7 (3.9)	26.0 (4.0; n = 243)
History of hypertension, %	63.5 (3365/5302)	78.2 (190/243)
History of diabetes, %	2.8 (121/4358)	13.3 (27/203)
Current smoking, %	27.4 (1453/5302)	53.9 (131/243)
Total cholesterol, mmol/L, median (IQR)	6.1 (5.4–6.8; n = 5237)	6.3 (5.7–7.1; n = 239)
Triglycerides, mmol/L, median (IQR)	1.2 (0.9–1.6; n = 5236)	1.4 (1.0–2.0; n = 239)
Haemoglobin A1c, %, median (IQR)	4.8 (4.5–5.1; n = 5231)	5.0 (4.7–5.5; n = 240)
Plasma inflammatory biomarkers, median (IQR)		
CRP (mg/L)	1.4 (0.7–2.8) (n = 5071)	2.1 (1.0–4.6) (n = 229)
Proneurotensin (pmol/L)	104.6 (75.7–148.0) (n = 4420)	108.0 (77.7–156.5) (n = 207)
Lp-PLAS_2_ (mass) (ng/ml)	255.0 (214.0–316.5) (n = 5154)	279.2 (224.9–345.1) (n = 236)
Lp-PLAS2 (activity) (nmol/min/ml)	44.0 (36.2–52.7) (n = 5158)	49.4 (40.6–59.7) (n = 237)

PAD; peripheral artery disease, CRP; C-reactive protein, Lp-PLA_2_ (activity and mass); lipoprotein-associated phospholipase A2, SD; standard deviation, IQR; interquartile range.

### Plasma biomarkers

**Figure 2:**
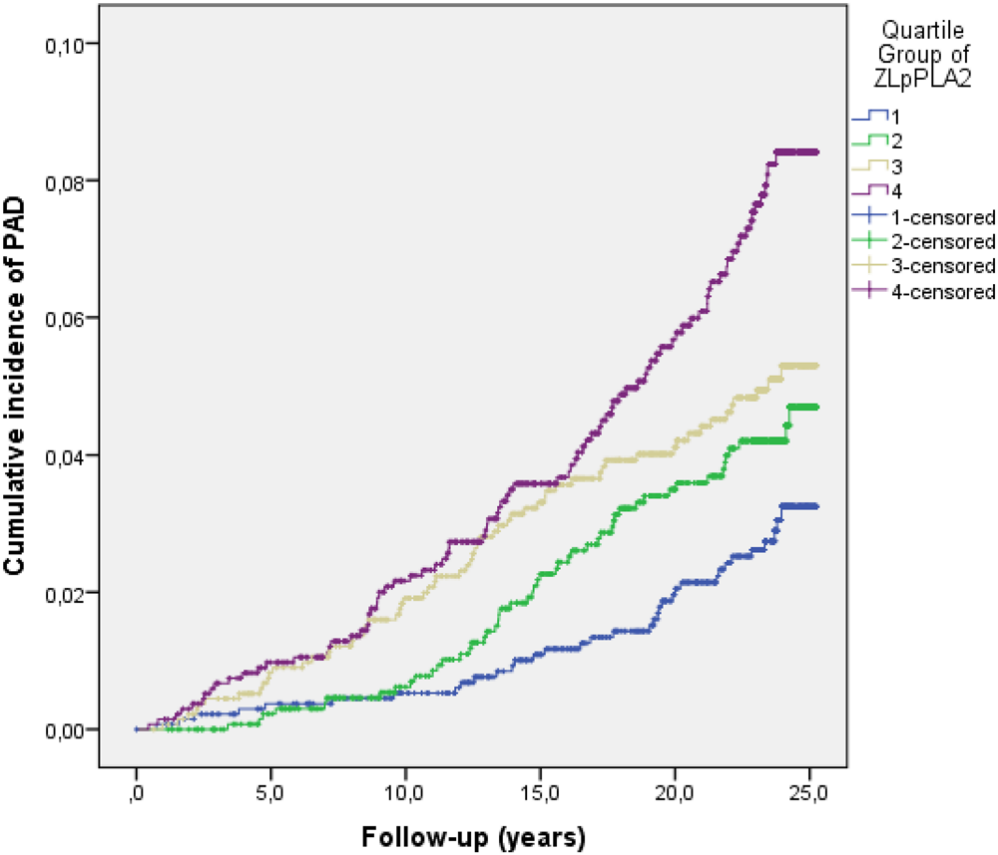
Cumulative incidence of PAD in relation to Lp-PLA_2_ activity stratified in quartiles.

Plasma biomarkers at baseline in individuals with and without incident PAD are shown in Table 1. When Lp-PLA_2_ activity was stratified to an ordinal variable in quartiles, an increased risk in cumulative incidence of PAD over the quartiles was found (p < 0.001). Cumulative incidence of PAD in the highest quartile was 2.6 times higher compared to the lowest quartile (Fig. 2). In the multi-variable adjusted analysis, Lp-PLA_2_ activity (HR 1.33; 95% CI 1.17–1.52), Lp-PLA_2_ mass (HR 1.20; 95% CI 1.05–1.37), and CRP (HR 1.55; 95% CI 1.36–1.76) were all independently associated with incident PAD (Table 2). When CRP was added as a covariate together with Lp-PLA_2_ (activity or mass), besides *age at study entry*, *sex, BMI*, *current smoking*, *diabetes mellitus*, *hypertension*, in the extended multi-variable analysis, Lp-PLA_2_ activity (HR 1.33; 95% CI 1.17–1.52; p < 0.001), Lp-PLA_2_ mass (HR 1.16; 95% CI 1.01–1.32; p = 0.038) and CRP (HR 1.36; 95% CI 1.18–1.58; p < 0.001) remained associated with incident PAD.

**Table 2:** Multi-variable adjusted hazards ratios for incident PAD in relation to plasma biomarkers.

Variable	PAD	p
	N = 244	
	HR* (95% CI)	
**Plasma inflammatory biomarkers**		
CRP (n = 5300)	1.55 (1.36–1.76)	<0.001
Proneurotensin (n = 4627)	0.94 (0.80–1.09)	0.41
Lp-PLAS_2_ (mass) (n = 5390)	1.20 (1.05–1.37)	0.008
Lp-PLAS_2_ (activity) (n = 5395)	1.33 (1.17–1.52)	<0.001

The following variables were entered in the multivariable analysis besides each respective plasma biomarker:

Age at study entry, sex, body mass index, current smoking, diabetes mellitus, hypertension.

*Hazard ratios (HR) were expressed per one SD increment of each respective log transformed plasma biomarker.

PAD; peripheral artery disease, CRP; C-reactive protein, Lp-PLA_2_ (activity and mass); lipoprotein-associated phospholipase A2, HR; Hazard Ratio, CI; Confidence interval.

## Discussion

The present longitudinal prospective study suggests that elevation of plasma Lp-PLA_2_ (activity), Lp-PLA_2_ (mass), and CRP, are all markers of subclinical disease susceptibility long time before diagnosis of PAD. This finding suggests that these biomarkers indicate subclinical PAD prior to onset of symptoms due to PAD. The association between Lp-PLA_2_ and incident PAD has previously been evaluated in three different cohorts, the Cardiovascular Health Study^20^, Multi-Ethnic Study of Atherosclerosis (MESA)^21^ and Atherosclerosis Risk in Communities (ARIC) study^22^. The Cardiovascular Health and ARIC study showed an association between Lp-PLA_2_ and incident PAD, whereas the MESA study showed no association. The negative findings in the MESA study^21^ were probably related to the fact that PAD was exclusively defined with repeated measurements of ankle-brachial index (ABI), resulting in inclusion of mainly asymptomatic PAD patients, whereas the other cohort studies used clinical endpoint data retrieved from hospital registries. Hence, it is likely that individuals that later develop symptomatic PAD as opposed to asymptomatic PAD may have been exposed to larger amounts of pathophysiological stimulators such as Lp-PLA_2_, promoting development of PAD. Plasma Lp-PLA_2_ (activity) and Lp-PLA_2_ (mass) were both strong predictors for incident PAD in the present study, since these biomarkers remained independently associated with incident PAD after adjusting for confounders in the extended multivariable analysis including the well established risk marker CRP^6,7^. The elevated Lp-PLA_2_ levels themselves, however, seem unlikely to be a primary causal factor for atherosclerotic disease according to phase III randomized controlled trials of inhibitors of Lp-PLA_2_, varespladib and darapladib^23^, and also to genetic studies using Mendelian randomization^24^. Instead, Lp-PLA_2_ levels appears to reflect an ongoing inflammatory process.

CRP is an established marker of systemic inflammation, vascular disease, and increased risk in PAD patients^6,7^ and the present study data showed that CRP, after adjusting for relevant confounders, was elevated already at baseline in patients developing symptomatic PAD, probably reflecting presence of subclinical atherosclerotic disease. Results from an interventional trial evaluating rosuvastatin (JUPITER)^25^ suggest that CRP may assist in risk stratifying healthy participants without hyperlipidemia for rosuvastatin treatment in primary prevention of atherosclerosis.

Increased plasma proneurotensin levels has been shown to be strongly associated with obesity^26^ and a predictor of cardiovascular disease in both genders and diabetes mellitus in women in two separate prospective cohorts^10,27^. The absence of association between proneurotensin and incident PAD, may be attributed to the finding that body mass index not was associated with incident PAD in the present study.

The MDCS cohort was originally designed to investigate the effects of diet on cancer risk^28^. A limitation of the study was that ABI not was measured at baseline to classify and exclude asymptomatic PAD individuals. Re-invitation of study participant survivors for detection of asymptomatic PAD by measurement of ABI would have been interesting to increase the total number of incident PAD individuals, perform subgroup analysis of factors associated with symptomatic and asymptomatic incident PAD, and increase power in the overall statistical analysis. A major strength of the present longitudinal study design, on the other hand, is the inclusion of healthy middle-aged individuals followed up for a median time of 23.4 years. Re-invitation would also have captured changes in risk factor status among study participants during this long period. During the last decade, the declining prevalence of smoking and the improved pharmacological treatment among individuals with cardiovascular disease might well have impacted the cumulative incidence of, above all, symptomatic PAD^29^. Another strength of this study was the high validity of symptomatic PAD, we could reliably confirm the PAD diagnosis in 98% of cases.

Lp-PLA_2_ mass and activity, but not CRP, have previously been found to be predictors of incident abdominal aortic aneurysm (AAA)^30^ in the same cohort during shorter follow up. CRP was the strongest predictor for incident PAD in the present study, and this discrepancy compared to the findings concerning AAA prediction may be interpreted as suggesting that PAD is a more inflammation-driven disease than AAA. Whereas the two diseases shared some similar risk factors in the two cohort studies, diabetes mellitus and hypertension were associated with incident PAD, but not with incident AAA. Actually, diabetes mellitus is a protective factor and reduces the incidence of AAA^31^. These differences in premorbid plasma biomarker profile highlight that PAD and AAA are different diseases with different pathophysiologies which should be analyzed and discussed separately.

In conclusion, plasma biomarkers Lp-PLA_2_ activity and mass were found to be useful markers of PAD risk during long-term follow-up together with the established biomarker CRP, implying that they might also be used as predictors for subclinical atherosclerosis and atherosclerotic disease.
